# Retraction: A new Mad2-interacting domain of Cdc20 is critical for the function of Mad2–Cdc20 complex in the spindle assembly checkpoint

**DOI:** 10.1042/BCJ20051914_RET

**Published:** 2026-06-03

**Authors:** Susanta Roychoudhury, Gourish Mondal, Rathindra N. Baral

**Affiliations:** Human Genetics and Genomics Division, Indian Institute of Chemical Biology, Kolkata-700 032, India

**Keywords:** cell division cycle 20 (Cdc20) domain, Mad2–Cdc20 interaction, nocodazole, polyhistidine motif, spindle assembly checkpoint, WD repeat

This article is being retracted from the *Biochemical Journal* at the request of the Editorial Board and its Chair. This action follows the receipt of a notification from a reader, alerting the Editorial Office to multiple similarities present within the paper as well as with Mondal et al. 2007 (DOI: 10.1093/carcin/bgl100), which has two authors in common.

Specifically:

- Multiple panels within Figure 8 appear to be duplicated. This includes:
  ◦ B/Mock with A/Wt (vertically flipped)
  ◦ Regions of A/GM5 with A/del-N
  ◦ Regions of B/Mock with C/Mock, B/del-N and C/del-N
  ◦ B/GM3 with C/GM5
  ◦ B/GM6 with C/GM4
  ◦ B/delN with C/del-N
  ◦ Regions of C/GM2 with C/del-C
- Multiple panels of Figure 8 overlap with panels from Figure 4B of Mondal et al. 2007 (DOI: 10.1093/carcin/bgl100)
  ◦ A/GM6 with -Noc/ + Cdc20 from Mondal et al.
  ◦ B/GM6 and C/GM4 with +Noc/ + Cdc20 from Mondal et al.
  ◦ B/del-C with +Noc/ + Cdc20 from Mondal et al.
- The Figure 7 IP and IP/EDTA bands for the lower Mad2 blot appear highly similar.

The authors have been contacted with regard to the concerns and the retraction. No raw data was available for the Figure 7 western blots, and the authors were unable to explain the similarities. Regarding the FACS diagrams listed above, the authors noted pixel-level differences, as well as differences in the size and distance of M1/M2/M3 texts from the scale bars, between similar images. Reasons for the similarities noted were not provided.

The Editorial Board has assessed all the information presented by the authors and serious concerns remain. The Board therefore stands by the decision to retract.

